# Examining racial identity invalidation and well‐being among Biracial adolescents using the identity capital model

**DOI:** 10.1111/jora.70084

**Published:** 2025-10-09

**Authors:** McKenzie N. Green

**Affiliations:** ^1^ Virginia Commonwealth University Richmond VA USA

**Keywords:** biracial, discrimination, racial identity invalidation, well‐being

## Abstract

Biracial Black–White adolescents report more psychological distress than most monoracial youth, but less is known about the factors that precede or protect Biracial youth from such distress. This study examines how racial identity invalidation (RII; the denial of a Biracial person's racial identity/belonging) relates to depressive symptoms and satisfaction with life (SWL) among 330 Biracial Black–White adolescents in the United States (67% boys; *M*_age_ = 14.8, *SD* = 1.5). Guided by the identity capital model, it also examines whether racial flexibility (e.g., shifting between different racial identities based on what race is valued in a social context) and personal authenticity moderate those associations. The analyses included two moderated moderation regression models, which showed that RII was associated with more depressive symptoms and less satisfaction with life. Significant interaction effects emerged, illustrating that racial flexibility and authenticity may be promotive and protective for Biracial Black–White adolescents. The two moderators, however, functioned differently for each indicator of well‐being (e.g., depressive symptoms vs. satisfaction with life). Implications for research and practice are discussed.

## INTRODUCTION

Biracial adolescents or youth who identify with two racial‐ethnic backgrounds are one of the fastest‐growing subgroups in the United States, with numbers expected to triple by 2060 (Vespa et al., 2018). Biracial Black–White youth (those with 1 biological Black parent and 1 biological white parent) comprise one of the largest subgroups of the young Biracial demographic (U.S. Census, 2023). Like monoracial youth (e.g., those with parents from the same racial‐ethnic group), adolescence marks a salient period of racial identity exploration and development for Biracial Black–White youth, who navigate a unique racialized experience as they defy the dominant ideology of racial essentialism or the belief that race is a singular and fixed construct (Renn, 2008). During adolescence, Biracial Black–White youth specifically face new social experiences and challenges that prompt them to continuously reflect, negotiate, and explain the meaning and significance of race in their lives. As such, changes in racial identity are seen as a normative and potentially protective part of Biracial development (Nishina et al., 2010). Scholars specifically theorize that *racial flexibility* or the tendency to emphasize a racial identity (e.g., Black, white, or Biracial) that is most valuable in a specific social situation or context can be a pathway to well‐being for Biracial people (Shih et al., 2019). Please note that white is intentionally not capitalized throughout the text in alignment with the belief that capitalization should be reserved for groups that have been distinctly harmed and marginalized by racist systems of power that privilege whiteness and white people (see Bauer & Ellis, 2023; Stewart, 2023).

Racial flexibility (originally referred to as a protean identity by Rockquemore and Brunsma (2002) and as racial malleability in social psychology (Pauker et al., 2018)) is akin to code switching. The practice may be particularly helpful in protecting Biracial young people from the harmful effects of *racial identity invalidation (RII)*, which is the rejection of a person's racial identity or belonging to a racial group (Albuja et al., 2018; Franco & Franco, 2016). However, if this flexibility is performed at the insistence of others and not because it feels authentic to the Biracial adolescent, then it could pose a psychological risk (Lusk et al., 2010), but this proposition has not been empirically explored. The current study begins to address this gap by examining relationships between RII, racial flexibility, authenticity, and well‐being among Biracial Black–White adolescents.

### Understanding biracial identity through the identity capital model

*The identity capital model* (ICM; Côté, 2016) provides a useful theoretical framework for examining identity flexibility and how it impacts Biracial Black–White adolescents. The ICM is a multidimensional theoretical perspective that builds heavily on Erikson's (1968) seminal work on identity formation during adolescence as well as symbolic interactionism and late modernity to outline the ways in which people, especially adolescents, in contemporary society “develop, organize, and execute a portfolio of identity‐based resources (e.g., identity capital)” to meet the demands of their social environments (p. 4). The model contends that the destabilization of socio‐historical and cultural norms has opened the door for a more individualized life course to unfold, where youth have more room to negotiate their identities with respect to certain risks and opportunities. As such, the ICM perceives identities in contemporary society as tools or *capital* that can be actively or passively managed to maximize social belonging. This occurs by mastering the symbolic codes (e.g., the language, habits, attitudes, expectations) of one's social groups. However, societal changes ultimately mean that young people have “more *freedoms from* normative constraints, not *freedoms to* pursue activities independent of systemic barriers” (p. 12). Côté further explains that this freedom only exists because of a lack of “normative structure,” which can still pose serious social challenges. Consequently, identity capital is vital to helping young people thrive in a society that simultaneously lacks normative structures and yet still carries the residual burdens of historical structural barriers (Côté, 2016).

The juxtaposition between having more freedom but less structure is especially evident in the racial lives of Biracial Black–White adolescents. Through the lens of the ICM, one can see how the abolition of slavery and the overturning of anti‐miscegenation laws in the Loving vs. Virginia case in 1967 sparked socio‐historical changes that disrupted existing racial structures and lines in the United States. Prior to these events, the racial identity of Biracial Black–White people was legally and socially determined by the “one‐drop rule” also known as the hypodescent principle (Root, 2003). This principle is a racial classification system that assigns individuals with any Black ancestry to the Black “lower” racial group, reinforcing historical racial hierarchies. Thus, for many decades, Biracial Black‐White people largely saw themselves and were seen by others solely as Black as this was the social and legal norm (Hollinger, 2005). That norm, however, has destabilized with an increase in interracial partnerships and advocacy efforts (Ho et al., 2017; Roth, 2005), granting the younger generation of Biracial Black–White people more *choice* in how they define themselves (Brunsma et al., 2013).

Rockquemore and Brunsma (2002) presented a typology of four racial identity options that are available to Biracial Black–White people in more contemporary social times: (a) singular identity where they may identify as exclusively Black or white, (b) border identity where they identify as exclusively Biracial, (c) protean identity where they “may move fluidly between black, white, and/or Biracial identities calling forth whatever racial identity seems situationally appropriate in any particular interactional setting and cultural community,” and (d) a transcendent identity where they reject racial labels altogether (Rockquemore et al., 2009, p. 338). Notably, protean identities were not commonly reported during the early 2000s (Rockquemore & Brunsma, 2002), but research on racial flexibility demonstrates that it has become increasingly more frequent in recent years (Echols et al., 2018; Lou et al., 2011). One reason for this trend may be the deviations in how racial flexibility/protean identity has been assessed. Seminal studies (Brunsma, 2006; Lusk et al., 2010; Rockquemore, 1998) primarily treated flexibility as an *identity* based on Rockquemore typology (e.g., participants picked between identifying as Black, Biracial, white, *or* a flexible combination of them), whereas more recent studies assess racial flexibility as an identity‐based *strategy* acknowledging that someone can see themselves as Biracial, but still engage in flexibility as a way to thrive socially (Cardwell et al., 2023; Sanchez et al., 2009). Thus, racial flexibility may now be best understood as a “constant process of *doing* race,” where Biracial people deploy different racial identities based on their social, cultural, and political realities (Brunsma & Delgado, 2008, p. 337).

### Racial flexibility and RII

The phenomenon of racial identity flexibility is aligned with identity capital outlined in the ICM, such that Biracial youth learn about their racial fluidity as well as the symbolic codes of their racial groups and contexts through observation and socialization (Johnson, 2024). They may subsequently use this information to strategically manage their racial presentation and behaviors based on the demands of their social lives (Côté, 2016). The ability to practice racial flexibility, which was only made possible through the destabilization of color lines in the United States, may protect Biracial youth from being targets of hostility or aggression in various racial contexts (Shih et al., 2019). However, this destabilization merely grants Biracial Black–White youth *freedom from* the narrow constraints of the hypodescent rule but not necessarily the *freedom to* live out a racial life that is free from the residual burdens of the hypodescent rule and anti‐Blackness (Côté, 2016). The absence of more strict color lines positions Biracial Black–White youth in a unique social position where some people may see them as either Black, Biracial, *or* white whereas others may see and accept them as Black, Biracial, *and* white (Roberts et al., 2022). The lack of a universal or essentialist classification for Biracial Black–White young people thus creates the space for RII to manifest.

RII is one of the most common forms of interpersonal racial discrimination among Biracial Black–White people (Campion, 2019; Franco et al., 2016; Norman et al., 2023). RII is the “misalignment between an individual's self‐defined racial identity and the way that others perceive them within a particular context” (Franco & O'Brien, 2018, p. 116). This invalidation can come from a variety of sources (e.g., peers, teachers, family, strangers) and include rejection of a person's identity (e.g., telling a Biracial teenager who identifies as Black they are “not really Black”) or imposing a racial identity onto a Biracial person (e.g., telling a Biracial teenager “You think you are Biracial but really you are just Black”). Both forms of invalidation are rooted in antiquated classifications and essentialist ideologies of race as singular and mutually exclusive. Notably, the ways in which Biracial people are perceived racially by others are ever‐changing and shift largely based on historical, social, political, cultural, and physical factors (Albuja et al., 2023; Ho et al., 2011; Oliver et al., 2024). The failure to navigate and master these ever‐changing social expectations can leave Biracial people vulnerable to social isolation, hypervigilance, and low self‐esteem (Nadrich, 2024; Sonoda & Garrison, 2023), all of which have consequences for their psychological well‐being. Scholars have specifically linked RII with symptoms of depression, anxiety, stress, and negative affect (Franco, Durkee, et al., 2021, Franco, Toomey, et al., 2021; Oh et al., 2024). This study will examine whether general experiences of RII relate to depression symptoms during adolescence specifically. This investigation is especially important as RII is not well understood among the younger demographic. This gap is concerning as the literature on social belonging (Cohen, 2022; Delelis, 2023) and racial discrimination (Cave et al., 2020; Huynh & Fuligni, 2010) suggests that RII may be particularly harmful during adolescence as it is a developmental period where conformity, socioaffective sensitivity, and peer influences are particularly salient (Laursen & Veenstra, 2021). Specifically, racial discrimination and peer prejudice can heighten feelings of not belonging – a critically important social need for adolescents – which, if unmet, can lead to internalizing symptoms such as depression and suicidal ideation (Barzeva et al., 2020; Boyd et al., 2024). RII warrants further investigation among Biracial adolescents, as it is a form of racial teasing that may spark feelings of not belonging and, in turn, worsen their mental health.

Racial identity flexibility may be a powerful buffer against RII and psychological distress as it can offer Biracial youth refuge from invalidation in different contexts and help them sustain a sense of belonging (Kramer et al., 2015; Shih et al., 2019). However, the research on the potential benefits of racial flexibility (which has been done primarily with adults) has been inconsistent. Some research has suggested that higher levels of flexibility are associated with worse psychological well‐being (Sanchez et al., 2009). The inconsistencies in the association between flexibility and well‐being in the literature may be due to the complexity of the phenomenon of malleable identification and the reasons behind how and why it is practiced. Like other forms of identity capital, racial flexibility may be rooted in a passive disposition where a person lets others make decisions about their racial identity to avoid resistance and conflict. Comparatively, flexibility can be rooted in more of a conscious disposition in which a person takes more agency over their racial identity and how they deploy it. The ICM implies that the latter approach is likely more adaptive as it is more authentic to the individual (Côté, 2016).

### The role of authenticity

Authenticity – or the sense of being the “real me” – is a central predictor of psychosocial well‐being for adolescents. During this developmental stage, youth feel an increased urge to behave in ways that align with their values and true sense of self (Alchin et al., 2023). Adolescence is simultaneously marked by the differentiation of multiple selves, where youth explore and understand the multiplicity of themselves across different roles and social relationships (Harter et al., 1997). Thus, “having multiple identities is normal and what is important for flourishing across adolescence is the degree to which a person's identities are becoming integrated (e.g., represent one's own values and beliefs), rather than introjected (reluctantly adopted)” (Alchin et al., 2023, p. 280). In line with this perspective, Lusk et al. (2010) theorize that authenticity likely plays a significant role in whether racial identity flexibility is protective or healthy for Biracial Black–White people. From a developmental perspective, an agentic or authentic approach to racial flexibility may be more challenging for younger adolescents to adopt as they report being more influenced by others, especially their peers, than mid‐to‐late adolescents (Blakemore, 2018). However, this has not been empirically explored.

### The current study

In comparison to their monoracial counterparts, Biracial Black–White adolescents experience disparate levels of psychological well‐being (e.g., more depressive symptoms, substance use, suicidality) (John Hopkins, 2023; Miller et al., 2019; Nishina & Witkow, 2020; Oh et al., 2023). Separate bodies of scholarship with Biracial people across the lifespan have indicated that RII is a salient risk factor for mental health, whereas racial identity flexibility may be an important protective factor. The current literature, however, remains limited in several ways including (a) adolescents and developmental considerations are largely absent, (b) the association between RII and flexibility has not been explored in the same model(s), and (c) the findings on flexibility are mixed with some research positioning it as a risk factor associated with more psychological distress. The goal of the present study was to push this scholarship forward by examining three research questions: (1) Is there an association between lifetime experiences of RII and well‐being (e.g., depressive symptoms and satisfaction with life) among Biracial Black–White adolescents? (2) Does racial identity flexibility moderate the respective associations between RII and well‐being? and (3) Does authenticity moderate the conditional influence of identity flexibility on the relationship between RII and well‐being? Based on prior research, I expect that RII will be associated with more depressive symptoms and less satisfaction with life. Guided by the ICM and Lusk et al. (2010), I anticipate that racial identity flexibility may buffer or moderate the association between RII and well‐being, but this may only be true for youth who report higher levels of authenticity. These findings make a unique contribution to the literature by addressing the identified gaps and providing a more nuanced theoretical understanding of whether and under what conditions racial flexibility – conceptualized as a form of identity capital – is advantageous for Biracial youth. This research highlights the possibility that outcomes associated with racial flexibility may vary depending on (a) developmental stage and (b) broader individual characteristics (e.g., authenticity). Such insights have important implications for determining whether, and in what ways, evidence‐based programs focused on racial flexibility and identity development should be designed and implemented for Biracial adolescents.

## METHODS

### Participants

Participants included 330 Biracial Black–White adolescents that ranged from ages 12 to 17 years old (*M* = 14.82, *SD* = 1.51) with 40.6% of the participants falling in the early adolescence range (12–14) and the remaining 59.4% falling in the mid‐to‐late adolescence period (15–17). About 67.3% of the participants were cisgender males with the addition of one transgender male participant, whereas the rest were cisgender females (32.4%). Many of the adolescents in the sample (92.4%) were heterosexual and the remaining were bisexual (3.9%), gay or lesbian (3.3%), and pansexual (0.3%). More than half of the youth (68.5%) had white mothers and Black fathers, which is consistent with the U.S. interracial marriage rate (Livingstone & Brown, 2017). Participants also reported on ethnicity among their parents. Many Black parents had African American (75.8%) or African (16.4%) ancestry, and the rest were reported as Afro‐Latinx (6.2%), Afro‐Caribbean/West Indian (1.7%), and Multiethnic (1.5%). Additionally, participants reported that their white parents were English (40.9%), Irish (11.8%), French (10.6%), German (9.7%), Italian (7.9%), Scottish (6.7%), Polish (4.5%), Multiethnic (3.6%), unreported (3.1%), or something else (1.2%). About 49% of mothers and 45% of fathers held a bachelor's or graduate degree. Youth also reported their living situations, where many lived in a home with both of their biological parents (80.7%) or in separate homes with each of the parents part‐time (12.8%). Participants lived in the southeastern (40%), northeastern (12%), midwestern (17%), and western (31%) regions of the United States. Additionally, zip codes were also reported, which were used to record neighborhood‐level characteristics using social mobility data from the U.S. Census Bureau (Mast & Din, 2021). According to the data, it was found that, on average, youth lived in neighborhoods where the poverty rate was 19%, the average annual household income was $56,157.76, and the fraction of non‐White residents was 40%.

### Procedure

Data come from a national cross‐sectional survey that examined racial identity, racial socialization, and well‐being among Biracial Black–White adolescents living in the United States. Recruitment occurred through a purposive sampling technique where social media advertisements were used to reach parents of Biracial youth. Parents were instructed to follow a link to an eligibility questionnaire on Qualtrics where a PDF copy of the parental permission form was displayed. The parent's permission was provided for their adolescent's participation by clicking “next” on the webpage and then instructed to allow their child to complete the remaining questions individually. Then, the youth participant was given a PDF copy of the assent form by clicking “next” on the web page. This indicated their assent to participate. After assent was given, adolescents continued with the eligibility questionnaire. Eligibility criteria included participants between the ages of 12–17 years old, one biological monoracial Black and one biological monoracial White parent and living in the United States with at least one of their biological parents. Data quality was protected by instructing eligible participants to provide an email address where the principal investigator could email an anonymous link to the official survey via Qualtrics. The survey took roughly 35 min to complete. Three participants were excluded from the data analysis due to failing two or more attention checks that were implemented throughout the survey (Curran, 2016). A $10 electronic gift card was given as compensation to participants. Data collection began during the spring of 2021 and concluded during the winter of 2022. The study protocols were approved by the Institutional Review Board.

### Measures

#### Racial identity invalidation

The behavioral invalidation subscale of the RII scale (Franco & O'Brien, 2018) was used to measure behavioral invalidation among the participants and was the only measure of RII used in the survey. The subscale consists of four items (e.g., “Because of the way I speak, others deny my racial group membership”) that are answered based on a 6‐point frequency scale (1 = Never happened to me to 6 = Happened to me more than 10 times). Notably, this scale does not ask respondents to specify the race of the perpetrator(s) of RII or the nature of their relationship to the perpetrator(s). Higher mean scores of the items represent more frequent experiences of RII. The behavioral invalidation subscale demonstrated sufficient internal consistency in the current sample (*α* = .81).

#### Racial identity flexibility

The flexibility of participants' racial identity was assessed using five items (e.g., “I often identify more with one racial identity than another depending on the race of the person I am with”) on a 6‐point scale (1 = Strongly disagree to 6 = Strongly agree). Sanchez et al. (2009) created the items to capture the degree to which Biracial people change their racial identification across situations, activities, and time. A higher mean score of the items indicates greater identity flexibility. The same items have been used in prior studies with Biracial and Multiracial populations and demonstrated adequate reliability (Cardwell et al., 2023), as they did in the present sample (*α* = .78).

#### (In)authenticity

The authenticity of participants was measured with a modified version of the authenticity scale (Wood et al., 2008). Participants responded to 8 items that were created to capture multiple aspects of authenticity, including the degree to which participants feel out of touch with themselves (e.g., “I feel very out of touch with the ‘real me.’”) and are impacted by external influences (e.g., “I usually do what other people tell me to do”). Participants responded to the items on a 7‐point scale (1 = does not describe me at all and 7 = describes me very well), with higher scores representing more inauthenticity and lower scores indicating more authenticity. Although the items were designed to be used across 2 distinct subscales (e.g., self‐alienation and accepting external influence), they were all included in one‐dimensional scale in the current study to simplify the models (described below). This decision was made based on the fact that both aspects of authenticity impact psychological well‐being similarly (Lutz et al., 2023; Wood et al., 2008). The interitem reliability for the 8 items was also within an acceptable range (*α* = .89).

#### Depressive symptoms

Symptoms of depression were assessed using the depression subscale of the depression, anxiety, and stress scale (DASS‐21; Henry & Crawford, 2005). The subscale consists of 7 items that ask participants how frequently they experienced symptoms of depression over the prior 2 weeks (1 = Did not apply to me at all to 4 = Applied to me most of the time). Sample items include “I felt that life was meaningless” and “I felt that I wasn't worth much as a person.” A mean score was created, with low scores indicating fewer depressive symptoms. The scale has been proven reliable among a racially diverse sample of Black and Multiracial Black adolescent boys (*α* = .90; Del Toro et al., 2019) and Black college students (*α* = .84; Norton, 2007). It also demonstrated strong internal consistency in the current sample (*α* = .83).

#### Satisfaction with life

The Satisfaction with Life Scale (SWLS; Diener et al., 1985) was administered to assess the degree to which participants were satisfied with their lives. The SWLS consists of 5 items (e.g., “In most ways my life is close to my ideal”). Participants indicated how much they agreed and disagreed with each statement using a 7‐point scale (1 = Strongly disagree to 7 = Strongly agree). The SWLS has been proven to be psychometrically sound within adolescent samples (Jovanović, 2016), which remained true for the current sample (*α* = .81).

#### Covariates

Age, gender (coded as 0 = female, 1 = male), and maternal and paternal education attainment were included as covariates in both models as they have been associated with mental health in prior research (Willroth et al., 2021; Yoon et al., 2023; Zhu et al., 2019). Phenotype (coded as 1 = I look Black and most people assume I am Black, 2 = my physical features are ambiguous, people assume I am a person of color mixed with another race, 3 = my physical features are ambiguous, people question what I am and their assumptions frequently change, and 4 = I physically look white; I could “pass” as white) and geographic region may impact experiences of RII, racial flexibility, and mental health to varying degrees (Bijou & Colen, 2022; Brunsma & Rockquemore, 2001; Gonlin, 2022), so those were included as covariates as well.

### Data analysis plan

**FIGURE 1:**
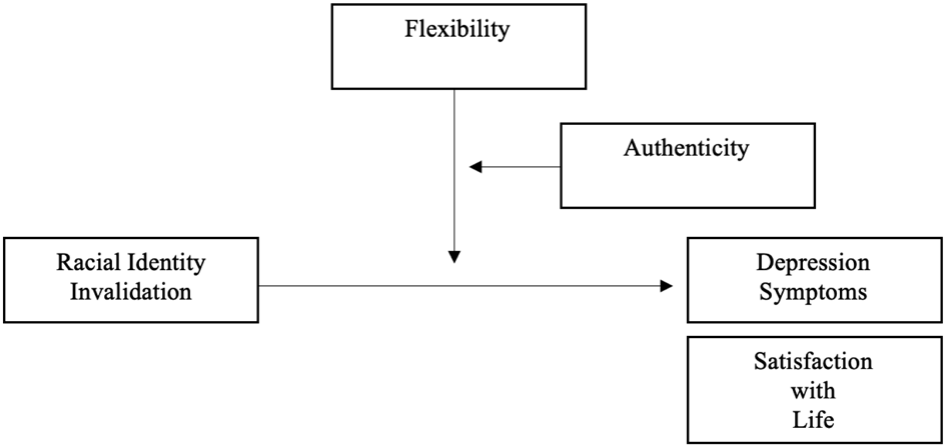
Moderated moderation of the effect of racial identity invalidation on depression symptoms and satisfaction with life by racial identity flexibility depending on authenticity.

All analyses were conducted in SPSS Version 28. First, descriptive statistics were run to explore the means, standard deviations, normality, and bivariate associations among the study variables. Participants responded to all items, so there were no issues with missing data. Then, for the primary analyses, two moderated moderation regression models were conducted using Model 3 of the Hayes PROCESS Macro for SPSS (Hayes, 2021). The models were identical with the exception of the outcome variable being either depressive symptoms or satisfaction with life (see Figure 1). In both models, the focal predictor (X) was RII, the primary moderator (M) was racial flexibility, and the secondary moderator (W) was authenticity. The variables were all mean‐centered prior to analysis. Consistent with the research questions, the goal was to determine whether authenticity strengthened or weakened the moderating role of racial flexibility in the associations between RII and well‐being. Significant interactions were probed using simple slope analyses (Preacher et al., 2006).

## RESULTS

### Descriptives

There were no issues with missingness of data and the skewness or kurtosis values all inside the range of normality (±1.0). The correlations between the study variables are presented in Table 1. On average, the adolescents experienced RII two to four times in their lifetimes (*M* = 3.14 out of 6), but there was a large degree of variation in those reports (*SD* = 1.05). This was likely due to differences based on age as the mid‐to‐late adolescents (*M* = 3.29) reported more RII than the early adolescents (*M* = 2.94); *t*(277) = −2.95, *p* = .003. Participants also reported a fair amount of racial flexibility (*M* = 4.22 out of 6; *SD* = .85) and inauthenticity (*M* = 3.54 out of 7; *SD* = 1.16). The adolescents appeared to be generally psychologically well with low to average reports of depressive symptoms (*M* = 1.82 out of 4; *SD* = .52) and moderate levels of satisfaction with life (M = 4.16 out of 7; *SD* = 1.18), with early adolescents reporting more satisfaction (*M* = 4.32) than mid‐to‐late adolescents; *t*(310) = 2.13, *p* = .03. No other developmental differences were present. The bivariate relationships were consistent with prior research and hypotheses, with RII being linked to more depressive symptoms and less satisfaction with life. RII was also associated with higher levels of racial flexibility and more authenticity. The descriptive statistics supported moving forward with the primary models.

**TABLE 1:** Correlations among study variables (*n* = 330).

Variable	1	2	3	4	5	6	7	8	9	10	11
1. Racial identity invalidation											
2. Flexibility	.42**										
3. (In)authenticity	−.18**	−.11									
4. Depressive symptoms	.13*	−.16**	.47**								
5. Satisfaction with life	−.35**	−.06	.34**	−.06							
6. Age (cov)	.21**	.12*	−.11*	.03	−.12*						
7. Gender (cov)	−.21*	−.26**	.09	.05	.19**	−.05					
8. Maternal education	−.08	.13*	−.20**	−.29**	.16**	.02	−.10				
9. Paternal education	−.05	.28*	−.11*	−.26**	.14*	.01	−.07	.43**			
10. Phenotype	−.05	−.10	.00	−.14**	−.01	−.04	.24**	.05	.02		
11. Region	.13*	.14**	−.02	−.05	−.01	.10	−.11	.14*	−.01	−.18**	

*Note*: Higher scores of inauthenticity indicate less authenticity.

* *p* < .05;

** *p* < .001.

### Model 1: RII, Flexibility, (In)authenticity, and Depressive Symptoms

**FIGURE 2:**
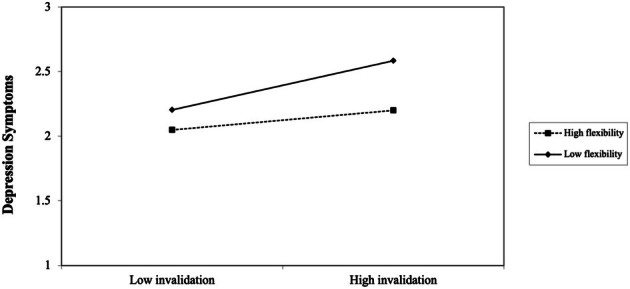
Racial Flexibility moderating the relationship between racial identity invalidation and depressive symptoms.

The first model (see Table 2) accounted for 40% of the variance in depressive symptoms *R* ^*2*^ = .40, *F*(13, 314) = 16.22, *p* < .001. As indicated in the table, the covariates of paternal education and phenotype were significantly associated with depressive symptoms such that participants who had fathers with less education and who had more Black or ambiguous phenotypes reported more depression symptoms than their counterparts. For main effects, RII (*B* = .14, *p* < .001) and inauthenticity (*B* = .21, *p* < .001) were significantly and positively associated with depressive symptoms. Comparatively, racial flexibility was associated with fewer depressive symptoms, *B* = −.13, *p* < .001. The two‐way interaction between RII and racial flexibility was also significant, *B* = −.05, *p* < .05. The simple slope analysis revealed that RII was associated with depressive symptoms for Biracial adolescents who reported high (1 SD over the mean) and low (1 SD below the mean) levels of flexibility. However, as depicted in Figure 2, youth with low flexibility reported more depressive symptoms (*B* = .19, *p* < .001) in the presence of low and high amounts of RII than youth with high flexibility (*B* = .08, *p* = .02). No other significant interactions emerged.

**TABLE 2:** Moderated moderation regression coefficients of racial flexibility and authenticity on racial identity invalidation and depressive symptoms among biracial adolescents (*n* = 330).

Variable	*B*	SE	*R* ^2^
Outcome: Depressive symptoms			.40***
Age (cov)	.04	.04	
Gender (cov)	.03	.05	
Maternal education (cov)	−.03	.03	
Paternal education (cov)	−.05*	.02	
Phenotype (cov)	−.12**	.04	
Region (cov)	−.02	.01	
RII	.14***	.03	
Racial flexibility	−.14***	.03	
(In)authenticity	.21***	.02	
RII × Racial flexibility	−.05*	.03	
RII × (In)authenticity	−.05	.03	
Racial flexibility × (In)authenticity	−.05	.04	
RII × Racial flexibility × (In)authenticity	−.02	.05	

Abbreviation: RII, racial identity invalidation.

* *p* < .05;

** *p* < .01;

*** *p* < .001.

### Model 2: RII, Flexibility, (In)authenticity, and Satisfaction with Life

**FIGURE 3:**
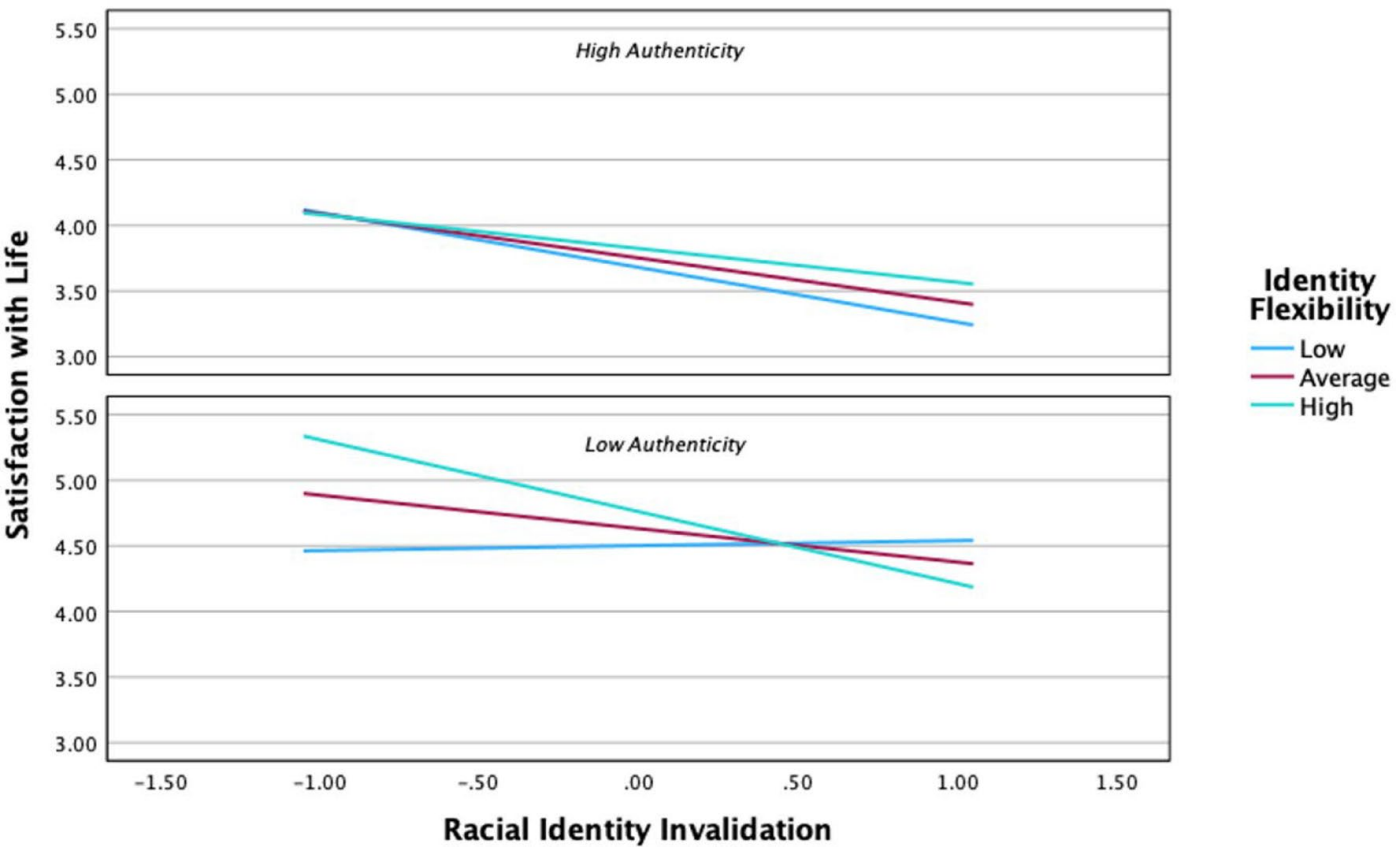
Racial flexibility and authenticity as moderators of the relationship between racial identity invalidation and satisfaction with life.

The second model (see Table 3) accounted for 31% of the variance in satisfaction with life, *R* ^2^ = .31, *F*(10, 318) = 10.64, *p* < .001. Gender and maternal education were significantly associated with SWL, such that being female and having a mother with a higher educational attainment were associated with more life satisfaction. For main effects, RII (*B* = *−*.29, *p* < .001) was significantly associated with less life satisfaction, whereas inauthenticity (*B* = .39, *p* < .001) was surprisingly associated with more. Comparatively, racial flexibility was not directly associated with satisfaction with life. However, a significant three‐way interaction emerged between RII, racial flexibility, and inauthenticity (*B* = −.18, *p* = .01). The findings indicate that flexibility moderates the association between RII and depression, but the findings differ based on inauthenticity (see Figure 3). For highly authentic adolescents, more racial flexibility was associated with more life satisfaction, particularly in the face of high amounts of RII.

**TABLE 3:** Moderated moderation regression coefficients of racial flexibility and authenticity on racial identity invalidation and satisfaction with life among biracial adolescents (*n* = 330).

Variable	*B*	SE	*R* ^2^
Outcome: satisfaction with life			.31***
Age (cov)	−.03	.04	
Gender (cov)	.35*	.13	
Maternal education (cov)	.20*	.06	
Paternal education (cov)	.08	.06	
Phenotype (cov)	−.11	.09	
Region (cov)	−.01	.04	
RII	−.29***	.07	
Racial flexibility	.12	.08	
(In)authenticity	.38***	.06	
RII × Racial flexibility	−.12	.07	
RII × (In)authenticity	.03	.06	
Racial flexibility × (In)authenticity	.04	.08	
RII × Racial flexibility × (In)authenticity	−.18**	.07	

Abbreviation: RII, racial identity invalidation.

* *p* < .05;

** *p* < .01;

*** *p* < .001.

The opposite was true for adolescents who reported low authenticity as high racial flexibility amid high RII corresponded with the lowest levels of life satisfaction for more inauthentic participants. Notably, when RII was low, participants who reported low authenticity and high flexibility reported being the most satisfied with their lives.

## DISCUSSION

The present study examined racial flexibility and authenticity as moderators of the relationship between RII and well‐being among Biracial Black–White adolescents. This examination extends prior research linking RII to mental health among adults to adolescents and sheds light on how it intersects with both racialized and general developmental processes (e.g., racial flexibility and authenticity) to influence their well‐being.

### Racial identity invalidation and psychological well‐being

RII was significantly associated with depressive symptoms among Biracial Black–White adolescents. This association is consistent with research that connects RII to depression and internalizing symptoms within Biracial adults (Campion, 2019; Franco et al., 2016; Norman et al., 2023). Linking RII to depressive symptoms during adolescence is an important advancement as it demonstrates how early RII begins and the potential consequences it may have on Biracial young people. This collective insight is valuable considering how salient social acceptance and affirmation are during this period of life (Alchin et al., 2023; Blakemore, 2018) and the disparate rates of depression reported by Biracial youth (Garcia et al., 2019; Miller et al., 2019; Subica & Wu, 2018).

The current study also propels the literature forward by linking RII to satisfaction with life, which to my knowledge, has not been done before. The findings specifically demonstrated that youth with more frequent experiences of RII were less satisfied with their lives than adolescents who experienced RII less frequently. These results illustrate that RII is not only linked with symptoms of psychological illness (e.g., symptoms of depression), but that it is also negatively associated with psychological thriving. Examining satisfaction with life as a dependent variable in the context of this study is aligned with calls from Positive Psychology and the State Model of Mental Health (Keyes & Martin, 2017) for researchers to position mental *illness* and mental *health* as separate continuums of equal importance. This perspective is rooted in the assumption that a “person can be free of mental illness without being fully healthy” (Keyes & Martin, 2017, p. 86). To date, much of the developmental research on well‐being has focused on the presence or absence of mental illness without much consideration of flourishing or satisfaction with life. Understanding and addressing mental illness during adolescence remains important; however, it is also imperative that scholars better understand life satisfaction during this period as it also holds powerful consequences for social adjustment, academic performance, and positive youth development (Lyons & Huebner, 2016; Oberle et al., 2011; Proctor & Linley, 2014). Ultimately, the study findings connect RII to both continuums of well‐being among Biracial Black–White adolescents, which has important research and practice implications that are discussed below.

### Demographic considerations

Several demographic covariates were integrated into the models, including youth age, gender, parental educational attainment, phenotype, and geographic region. Higher paternal educational attainment was associated with fewer depressive symptoms, whereas more maternal education was related to more life satisfaction. Parent education impacting mental health aligns with trends established in developmental science (Xiang et al., 2024). However, the fact that maternal and paternal education each impacted outcomes differently is intriguing and supports the need for research that incorporates reports of both parents' education as well as multiple indicators of wellness. Another surprising finding was that youth gender was not significantly associated with depression, which is not consistent with the extant developmental research, where monoracial females typically report more symptoms than monoracial males (Daly, 2022). However, Atkin et al. (2025) also found no evidence of gender differences in depression among their sample of 547 Multiracial young adults. Thus, it may be possible that gender plays less of a role in the etiology and/or reporting of depression among Biracial/Multiracial populations. This could be influenced by the fact that Biracial people are more comfortable with fluidity in their social identities and thus feel less pressure to conform to gender norms that increase risks of depression than monoracial people (Nielson et al., 2024). However, gender was significantly associated with satisfaction with life in the present study, where females were more likely to be content with their lives than males. This finding contributes to an ongoing and mixed body of research, with a recent meta‐analysis of 46 studies showing that “boys and girls report non‐significant differences” in life satisfaction (Chen et al., 2020, p. 2294). Again, it is possible that freedom from more typical gender norms is shaping patterns here, but this hypothesis warrants further exploration. Phenotype was the only other significant control variable, where Black phenotypes were associated with more depression symptoms. This could be due to a myriad of factors, such as participants with Black phenotypes being exposed to more instances of racial discrimination and colorism (Bozo et al., 2018), which increase risks of distress (Benner et al., 2018; Centeno et al., 2023).

### Racial flexibility and authenticity as moderators

The primary aim of this study was to examine racial flexibility and authenticity as potential moderators of RII on well‐being. Two moderated moderation models were employed to test an earlier theoretical proposition raised by Lusk et al. (2010) that racial flexibility would be the most protective for youth who perceived themselves as highly authentic. Based on this notion and the ICM, I suspected that racial identity flexibility may weaken the association between RII and well‐being, but this may only be true for youth who report higher levels of authenticity.

#### Depressive symptoms

The findings indicate that youth who practiced more racial flexibility (e.g., identifying with a certain race based on the race of the people they are around) reported fewer depressive symptoms than adolescents who reported less racial flexibility. This direct association is aligned with some of the previous literature indicating that racial flexibility may be psychologically beneficial (Shih et al., 2019) and contradicts other research suggesting that it is harmful (Sanchez et al., 2009). Consistent with the ICM, I suspect that racial flexibility was promotive for the adolescents when it enabled them to meet the ever‐changing demands of their social lives and “fit in” or feel a sense of belonging in a variety of settings (Côté, 2016). Racial flexibility also significantly moderated the association between RII and depressive symptoms, such that youth who reported below‐average levels of flexibility experienced the most depressive symptoms in the face of RII. Drawing again on the ICM, I believe RII may have been less impactful for youth that were racially flexible because they had another racial “home” of sorts that they could find safety in (Franco, Durkee, et al., 2021, Franco, Toomey, et al., 2021). Accordingly, the invalidation does not have as much power over them in comparison to participants who do not feel as comfortable shifting between different racial identities and thus likely only feel a sense of belonging to the group they are being invalidated from. This is likely especially true when the invalidation is coming from a member of their perceived in‐group. For instance, Franco and Franco (2016) found that RII was only associated with racial homelessness (e.g., perceived alienation from racial group members) for Biracial‐Black people if it was perpetrated by Black people. However, the measure of RII that was used in the present study did not ask respondents to specify the race of the person/people who invalidated them over their lifetime, so I was unable to incorporate that into the analyses. Nonetheless, racial flexibility did not completely shield Biracial youth from RII as those who reported above‐average levels still experienced elevated levels of depressive symptoms when RII was high. These results illuminate that racial flexibility is still a promising protective factor, further highlighting the devastating toll of RII on the well‐being of Biracial Black–White adolescents.

Adolescents who were more authentic were also less likely to experience depressive symptoms than those who reported greater levels of inauthenticity, which is also consistent with prior research (Alchin et al., 2023). Building on Lusk et al. (2010), I expected that higher levels of authenticity would strengthen the protective impact of racial flexibility on the relationship between RII and depression symptoms, but this was not the case. Additional research is needed to better understand these null findings, especially in light of the three‐way interaction between flexibility, authenticity, and satisfaction with life that emerged in model two.

#### Satisfaction with life

In contrast to the depression model, participants who were more authentic were significantly less satisfied with their lives than adolescents who reported greater levels of inauthenticity. This is relatively surprising considering the widespread acceptance of inauthenticity as psychologically risky, but there is some evidence that social capital (e.g., school connectedness and family support) impacts life satisfaction in adolescents more than individual capital (Calmeiro et al., 2018). However, there is a dearth of research that examines inauthenticity in relation to flourishing and thriving among adolescents (see Alchin et al., 2023 for a review), so this finding is still difficult to fully explain. Future research is warranted. Racial flexibility was also not directly associated with satisfaction with life. This finding was somewhat surprising given the literature linking racial flexibility to well‐being. However, similar to the trend observed with inauthenticity, it suggests that the singular effects of racial flexibility may depend on how well‐being is conceptualized (e.g., presence of distress vs. thriving). Moreover, the three‐way interaction was significant indicating that flexibility may moderate the influence of inauthenticity on life satisfaction. When Biracial adolescents perceived themselves as highly authentic, racial flexibility was protective in the face of high amounts of RII. However, racial flexibility was actually risky in the context of RII for adolescents who reported low authenticity, which aligns with Lusk et al.'s (2010) proposition that racial flexibility is most optimal for well‐being when it is performed authentically.

It is also noteworthy that life satisfaction was the highest (close to 5.5‐ on a 7‐point scale) among participants who reported low RII, low authenticity, and high racial flexibility. Thus, racial flexibility did not emerge as a main effect, but it nonetheless appears to have important implications for life satisfaction. These implications seem to vary depending on how frequently Biracial adolescents are exposed to invalidation and how in touch they feel with themselves. This could be due to a variety of factors, but I suspect that inauthenticity may actually make it easier for some Biracial youth to engage in racial flexibility. In turn, this flexibility could afford them greater social mobility and peer acceptance, ultimately enhancing their overall quality of life.

### Limitations and future directions

The study advances the developmental literature on Biracial youth and processes that impact their well‐being, but it must be interpreted with respect to several limitations. First and importantly, the data were cross‐sectional, meaning causal testing was not possible. Future studies should employ longitudinal designs that allow for a more in‐depth investigation of the temporal nature of the associations between RII, flexibility, authenticity, and well‐being. This gap is important to address as it could have meaningful implications for intervention and prevention development for Biracial adolescents. This is particularly true for understanding the best timing for such an intervention. The models in the current study were limited as they did not explicitly assess if RII, racial flexibility, or authenticity differentially impacted participants across different periods of adolescence. Future research should certainly interrogate age more critically. The measures used in this study were also limited to varying degrees. A lifetime measure of behavioral RII was used in the present study, which was sufficient, but it may be insightful to explore whether the timing of RII occurrences impacts the severity of its impact on well‐being. For instance, it is possible that more recent experiences are more detrimental than those that occurred during early childhood. The race and relationship of the perpetrator of RII will also be important to consider (Franco & Franco, 2016). However, to my knowledge, the only validated RII scale (Franco & O'Brien, 2018) was the one employed in this study, so qualitative investigations and further measurement development are needed to sufficiently explore these considerations. The measure of racial flexibility was also based on an overall agreement scale that implicitly positions flexibility to be somewhat stable (e.g., a person either has low, average, or high levels of flexibility). However, the salience of one's racial identity can vary quite considerably from day to day based on numerous ecological factors. Considering this, Ferguson et al. (2017) created the Cultural IDentity Influence Measure (CIDIM) to assess how people intentionally engage and disengage with aspects of their identities across time and context to meet the demands of their social environments (Ferguson et al., 2017). Future work should use the CIDIM to track flexibility across brief windows of time and consider whether it has the same promotive and protective benefits. Those studies could also still employ the racial flexibility measure that Sanchez et al. (2009) developed as a baseline measure of flexibility, as both conceptualizations are beneficial empirically and theoretically.

Additional research in this area should consider how adolescent demographics shape associations between RII, flexibility, authenticity, and well‐being. For instance, phenotype may shape whether and how youth can engage in racial flexibility, which could impact its consequences on their well‐being. White‐presenting Biracial Black‐White individuals have specifically reported being unwilling or unable to identify with their Blackness because of their phenotype (Pilgrim, 2021). This inherently limits the degree of flexibility they may engage in but also may limit the severity or frequency of RII occurrences as well. The nuance that phenotype alone adds to the conversation is just one example of how additional demographic characteristics (gender, age, ethnicity) warrant investigation to fully understand the relationship between RII, flexibility, authenticity, and well‐being. Additionally, scholars should remain mindful that racial flexibility may not be all *good* or *bad*. Racial flexibility appears to offer promotive and protective benefits for youth in the face of RII; it may also elicit social repercussions from monoracial counterparts (Albuja et al., 2018). Research should investigate how manifestations of racial flexibility may differentially impact the social reception of Biracial youth. Relatedly, racial flexibility among Biracial Black–White people should be investigated and interpreted as a process that is juxtaposed between oppression and privilege. Born out of oppression and monoracism, flexibility is an escape mechanism that can shield Biracial youth from discrimination like RII. However, it is also a unique social privilege that may enable Biracial youth to leverage their whiteness and/or Biraciality for social gains, which, if mishandled, could contribute to the oppression of monoracial minority people. Future research should therefore focus on the myriad of outcomes associated with flexibility for Biracial and monoracial populations.

## CONCLUSION

Biracial Black–White adolescents are one of the fastest‐growing youth populations in the United States but remain nearly invisible in developmental scholarship. The present study begins to address this gap by advancing the understanding of several risk, promotive, and protective processes among Biracial Black–White youth adolescents through the lens of the identity capital model (Côté, 2016). The findings support previous research that links RII to depression symptoms and spark a new line of work by demonstrating that it also relates to less satisfaction with life among Biracial Black–White adolescents. Both trends are noteworthy as they reveal the depths of how detrimental RII can be with respect to the spectrum of well‐being (Keyes & Martin, 2017). Drawing on the identity capital model (Côté, 2016), I also found that racial flexibility individually and collectively moderated the associations between RII and well‐being, albeit in slightly different ways. These collective findings hold meaningful implications for future research with Biracial adolescents. Furthermore, although RII is specific to Biracial youth, adolescents from diverse racial‐ethnic backgrounds experience identity invalidation (e.g., the “Acting white” accusation; Durkee et al., 2022), which has deleterious impacts on their mental health (Durkee & Gómez, 2022; Murray et al., 2012). The results from this study illuminate how valuable cultural and general forms of identity capital (e.g., codeswitching, authenticity, etc.) may be for the current generation of adolescents navigating the unique social terrain of *freedom from* but not *freedom to*. Additional research is needed, but these preliminary results suggest that adolescents may benefit from intervention and prevention programs that expand their identity capital.

## AUTHOR CONTRIBUTIONS

McKenzie N. Green was solely responsible for funding acquisition, data curation, project administration, conceptualization, methodology, formal analysis, and writing and reviewing the manuscript.

## FUNDING INFORMATION

Funding for the project was provided by the National Science Foundation, American Psychological Association, Society for Research in Child Development, and Society for Community Research and Action.

## CONFLICT OF INTEREST STATEMENT

There are no conflicts of interest to disclose.

## ETHICS STATEMENT

The data presented in this study were obtained through participants that fully consented to participation (with guardian approval) in study procedures that were approved by the Institutional Review Board at North Carolina State University (protocol number 20960) on June 4, 2020.

## Data Availability

The data that support the findings of this study are available upon request from the corresponding author. The data are not publicly available due to privacy or ethical restrictions.
